# Geographic variation in the treatment of non-ST-segment myocardial infarction in the English National Health Service: a cohort study

**DOI:** 10.1136/bmjopen-2016-011600

**Published:** 2016-07-12

**Authors:** T B Dondo, M Hall, A D Timmis, A T Yan, P D Batin, G Oliver, O A Alabas, P Norman, J E Deanfield, K Bloor, H Hemingway, C P Gale

**Affiliations:** 1 Leeds Institute of Cardiovascular and Metabolic Medicine, Leeds, UK; 2 The National Institute for Health Biomedical Research Unit, Barts Health, London, UK; 3 Department of Medicine, University of Toronto, Toronto, Ontario, Canada; 4 Department of Cardiology, The Mid Yorkshire Hospitals NHS Trust, Wakefield, UK; 5 National Health Service cardiac service user, West Yorkshire, UK; 6 School of Geography, University of Leeds, Leeds, UK; 7 National Institute for Cardiovascular Outcomes Research, University College London, London, UK; 8 Department of Health Sciences, University of York, York, UK; 9 The Farr Institute, University College London, London, UK; 10 York Teaching Hospital NHS Foundation Trust, York, UK

**Keywords:** NSTEMI, National Health Service, MINAP, Geographic variation, Clinical Commissioning Groups, Inequalities

## Abstract

**Objectives:**

To investigate geographic variation in guideline-indicated treatments for non-ST-elevation myocardial infarction (NSTEMI) in the English National Health Service (NHS).

**Design:**

Cohort study using registry data from the Myocardial Ischaemia National Audit Project.

**Setting:**

All Clinical Commissioning Groups (CCGs) (n=211) in the English NHS.

**Participants:**

357 228 patients with NSTEMI between 1 January 2003 and 30 June 2013.

**Main outcome measure:**

Proportion of eligible NSTEMI who received all eligible guideline-indicated treatments (optimal care) according to the date of guideline publication.

**Results:**

The proportion of NSTEMI who received optimal care was low (48 257/357 228; 13.5%) and varied between CCGs (median 12.8%, IQR 0.7–18.1%). The greatest geographic variation was for aldosterone antagonists (16.7%, 0.0–40.0%) and least for use of an ECG (96.7%, 92.5–98.7%). The highest rates of care were for acute aspirin (median 92.8%, IQR 88.6–97.1%), and aspirin (90.1%, 85.1–93.3%) and statins (86.4%, 82.3–91.2%) at hospital discharge. The lowest rates were for smoking cessation advice (median 11.6%, IQR 8.7–16.6%), dietary advice (32.4%, 23.9–41.7%) and the prescription of P2Y_12_ inhibitors (39.7%, 32.4–46.9%). After adjustment for case mix, nearly all (99.6%) of the variation was due to between-hospital differences (median 64.7%, IQR 57.4–70.0%; between-hospital variance: 1.92, 95% CI 1.51 to 2.44; interclass correlation 0.996, 95% CI 0.976 to 0.999).

**Conclusions:**

Across the English NHS, the optimal use of guideline-indicated treatments for NSTEMI was low. Variation in the use of specific treatments for NSTEMI was mostly explained by between-hospital differences in care. Performance-based commissioning may increase the use of NSTEMI treatments and, therefore, reduce premature cardiovascular deaths.

**Trial registration number:**

NCT02436187.

**Strengths and limitations of this study**

- This study evaluated care across a national healthcare service and used a clinical registry designed specifically to evaluate quality of non-ST-elevation myocardial infarction (NSTEMI) care.
- Advanced statistical techniques that allowed high-resolution analysis of combinations of pathways of care according to their eligibility and receipt were used.
- A detailed 10-year evaluation of receipt of care—few other national data sets can offer.
- Myocardial Ischaemia National Audit Project does not collect all cases of NSTEMI; thus, results of underuse of care interventions maybe underestimated.
- We used CCGs to investigate consistency in geographic unit performance over time, when they only recently have replaced Primary Care Trusts and may not have the same Cartesian boundaries.

## Introduction

Non-ST-elevation myocardial infarction (NSTEMI) is a leading cause of emergency hospitalisation in Europe and accounts for over 50 000 National Health Service (NHS) admissions per year.^1–3^ Mortality rates following NSTEMI are high, worse than that for ST-elevation myocardial infarction, and its incidence (which is already higher than STEMI)^4^ ^5^ is increasing with an ageing and multimorbid population.^6^ ^7^ However, clinical outcomes from NSTEMI may be improved through the use of guideline-indicated treatments including evidence-based pharmacological therapies and invasive coronary procedures.^8^

While hospitals are the cornerstone of the management of acute myocardial infarction, for many countries, treatments are determined by the local contracting of specialist services including that of ambulances, emergency departments and acute cardiac care. For the NHS of England, this is the responsibility of the 211 Clinical Commissioning Groups (CCGs) who work in partnership with hospitals, via Strategic Clinical Networks (SCNs) for National Institute for Health and Care Excellence support for commissioning for NSTEMI.^3^

Our earlier work found evidence for variation within and between the UK and Sweden in treatments and 30-day mortality from acute myocardial infarction.^1^ ^2^ ^9^ Such variation in cardiac services is estimated to cost the NHS £184 million.^10^ We have also shown that the majority of patients with acute myocardial infarction fail to receive at least one guideline-indicated treatment and that these missed opportunities were associated with cardiovascular deaths.^12^ For NSTEMI—the most common and vulnerable type of acute myocardial infarction—information concerning variation in guideline-indicated treatments is very limited.^13–17^ For the English NHS, there are no reports of how NSTEMI treatments vary according to CCGs, which leaves a gap in our knowledge as to how and where to focus efforts on reducing premature death from cardiovascular disease. Therefore, we used the UK heart attack register (Myocardial Ischaemia National Audit Project, MINAP) to conduct a 10-year study of the geographic variation in guideline-indicated treatments for NSTEMI.

## Methods

### Setting and design

**Figure 1:**
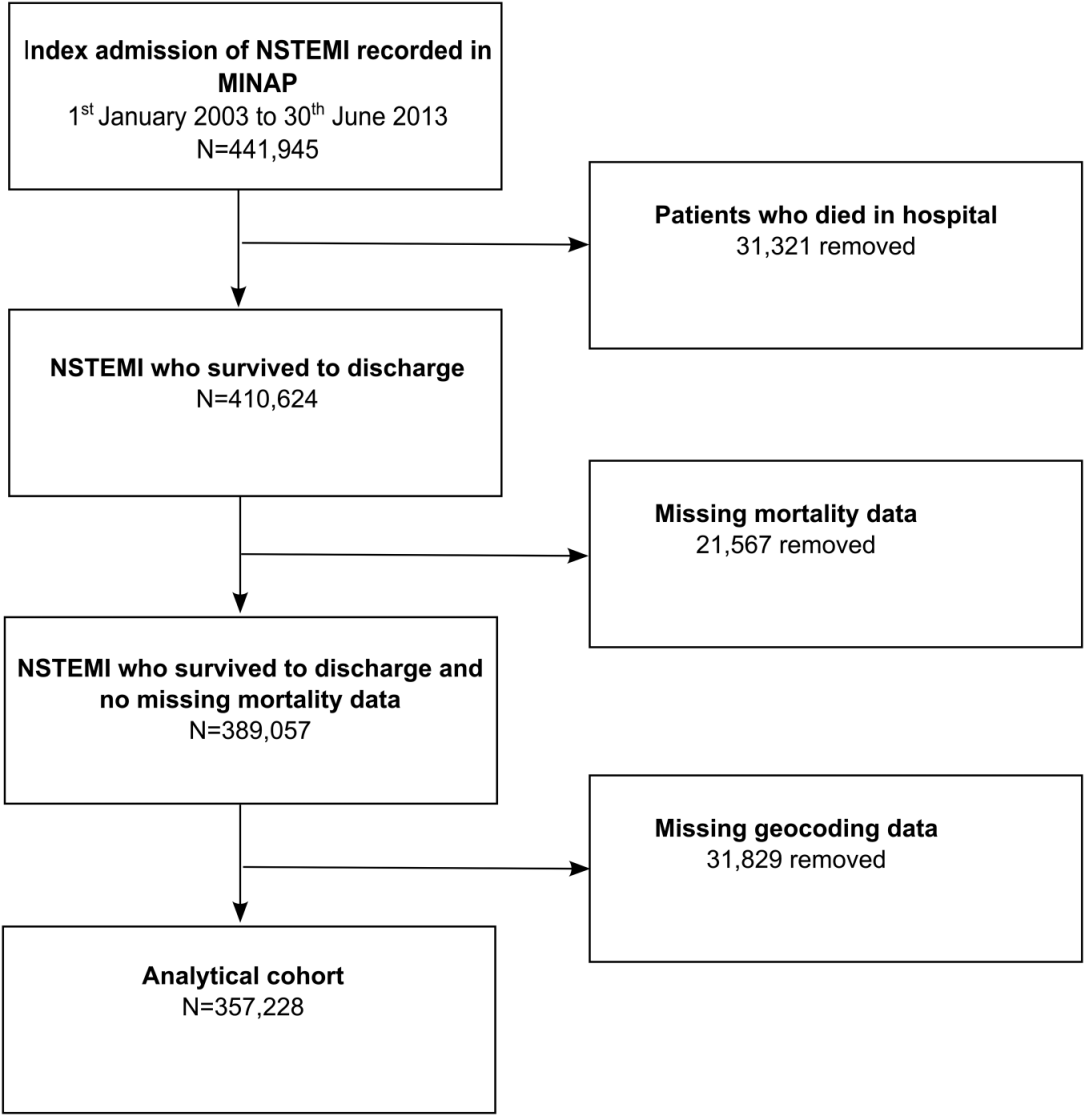
STROBE diagram of the derivation of the analytical cohort from the MINAP data set. MINAP, Myocardial Ischaemia National Audit Project; NSTEMI, non-ST-segment elevation myocardial infarction.

We included all NHS hospitals (n=232) in England which provided care for patients (n=357 228) aged over 18 years with NSTEMI between 1 January 2003 and 30 June 2013. The diagnosis of NSTEMI was based on guidelines from the European Society of Cardiology (ESC), American College of Cardiology and American Heart Association and determined at local level by the attending Consultant.^18^ For multiple admissions, we used the earliest record. Patient-level data concerning demographics, cardiovascular risk factors, medical history and clinical characteristics at the time of hospitalisation were extracted from MINAP, a comprehensive registry of hospitalisations for acute coronary syndrome in England and Wales, which was started in 2000 and is now mandated by the Department of Health.^19^ Details of MINAP have been described previously.^11^ The data flow for the derivation of the analytical cohort can be seen in figure 1.

### Patient involvement

No patients were involved in the design or implementation of the study. However, we involved a patient in the interpretation of the research and the writing of the research manuscript.

### Quality of care

We mapped iterative ESC guidelines for the management of NSTEMI and ESC Expert Consensus Documents to MINAP data to identify 13 guideline-indicated treatments as they became available over the study period.^20–25^ They included: the recording of an ECG, acute provision of aspirin, at hospital discharge the prescription of P2Y_12_ inhibitors, aspirin, β blockers among patients with left ventricular systolic dysfunction, ACE inhibitors (ACEis)/angiotensin receptor blockers (ARBs) among patients with left ventricular systolic dysfunction, aldosterone antagonists among patients with left ventricular dysfunction and either diabetes or heart failure without significant renal dysfunction, HMG-CoA reductase enzyme inhibitors (statins), and the use of early invasive procedures (coronary angiography), echocardiography, smoking cessation advice, dietary advice and enrolment into a cardiac rehabilitation programme.^8^ ^21^ We assessed the receipt of guideline-indicated treatments only for patients who were deemed eligible for each treatment according to the ESC guidelines.^20^ ^21^ ^23–25^ Patients were also classified as ineligible if a treatment was contraindicated, not indicated, not applicable, if the patient declined treatment as recorded in MINAP or if the patient was hospitalised prior to the time the treatment was recommended by the guidelines. See online supplementary tables S1 and figure S1 for information about how the ESC guidelines for the management of NSTEMI were mapped to MINAP data.

10.1136/bmjopen-2016-011600.supp1 Supplementary data

### Geographic units

We mapped each patient's treatment data, located by eastings and northings supplied by MINAP, to the April 2015 Geographic Information System CCGs layers (accessed from NHS England) and created choropleth maps to show the distribution of receipt of guideline-indicated treatments using ArcGIS V.10.2.2. We used class intervals with equal cut-offs for categorisation.

### Statistical analysis

We assessed the overall provision of guideline-indicated treatment by constructing composite scores for each patient. To do this, we divided the total number of treatments received by a patient by the total number of treatments that the patient was eligible for.^26–28^ Optimal care was defined as receiving all (up to 13) guideline-indicated treatments for which patients were eligible. We dichotomised the score as receipt of optimal care and non-receipt of optimal care (suboptimal care). Furthermore, the composite scores were categorised as high receipt (>79%), intermediate (40 to ≤79%) and low (≤40%) according to recognised cut-offs.^29^ ^30^ For optimal care and each of the 13 ESC guideline-indicated treatments in turn, we calculated the proportion of patients who received the treatment according to their location in a geographic unit. For example, for aspirin, this would be ‘did the patient, who was eligible to receive and had no contraindications, receive aspirin?’

We used percentages to describe categorical variables and means and SDs or medians, IQRs and ranges for continuous normally distributed and non-normally distributed variables, respectively. We used Spearman's correlation to assess the relationship between receipt of care in the earlier years (2003–2004) and receipt of care in the later years (2012–2013) by CCGs. We also represented temporal changes in optimal care among CCGs using Google Charts and motion maps.

To quantify variation within and between the geographic units, we used a four-level hierarchical Poisson model^31^ comprising patients nested within hospitals, nested within CCGs and nested within SCNs. The outcome (receipt of optimal care) was modelled as a count variable with a conditional Poisson distribution and all NSTEMI patients in the cohort as the exposure. The model incorporated patient-specific characteristics as fixed effects including demographics (sex, Index of Multiple Deprivation score and ethnicity), cardiovascular risk factors (diabetes, hypercholesterolaemia, hypertension, smoking status, asthma/chronic obstructive pulmonary disease (COPD) and family history of coronary heart disease), cardiovascular history (previous myocardial infarction, heart failure, percutaneous coronary intervention, coronary artery bypass grafting, angina, cerebrovascular disease and peripheral vascular disease) and the mini-GRACE risk score for predicted 6-month mortality.^32^ In addition, hospital, CCG and SCN random effects were included in the model to allow for clustering of patients within these levels. The intercept provided each patient's expected rate of guideline-indicated treatments (with a log transformation), adjusted for case mix. We used the interclass correlation (ICC) to quantify the proportion of variation in guideline-indicated treatments that was attributable to hospitals, CCGs and SCNs after adjustment for patient-specific characteristics. All analyses were performed using Stata V.13.

### Excess deaths

Multilevel accelerated failure time models were used to identify the association between missed guideline-indicated treatments and time to all-cause mortality. All models included a shared frailty term to account for clustering of patients within hospitals. Models were adjusted for case mix using the adjusted mini-GRACE risk score^32^ and for baseline patient characteristics including: previous history of myocardial infarction, angina, diabetes, hypertension, peripheral vascular disease, family history of coronary heart disease, asthma/COPD, hypercholesterolaemia and coronary revascularisation. Models were fitted on imputed data and estimates pooled over 10 imputations (see online supplementary table S2). In order to determine the potentially preventable deaths associated with suboptimal treatment for hospitals, the adjusted mortality risk (see online supplementary table S3) obtained from the multilevel accelerated failure time models was multiplied by the corresponding mortality rates and proportions of patients in the suboptimal treatment groups per hospital. The product was then multiplied by the total number of NSTEMI between 2003 and 2013 for each hospital (see online supplementary section 3).

## Results

Of 357 228 patients with NSTEMI (mean age 70.9 (SD 13.3) years), 63.1% (n=225 009) were men, the majority (93.1%, n=301 312) were white, one-third had angina and a quarter had previous myocardial infarction (table 1).

**Table 1:** Baseline characteristics, NSTEMI, 2003–2013

Characteristics	Cases n=357 228	Missing
Age, years; mean (SD)	70.9 (13.3)	504 (0.1)
Male	225 009 (63.1)	593 (0.2)
Deprivation according to IMD score		
1 (least deprived)	61 235 (17.2)	419 (0.1)
2	70 084 (19.6)	
3	74 842 (21.0)	
4	72 121 (20.2)	
5 (most deprived)	78 527 (22.0)	
Prior medical history		
Myocardial infarction	89 571 (25.1)	0*
Heart failure	22 581 (6.3)	0*
PCI	30 835 (8.6)	0*
CABG	26 021 (7.3)	0*
Angina	113 059 (31.7)	0*
Cerebrovascular disease	31 366 (8.8)	0*
Peripheral vascular disease	16 868 (4.7)	0*
Diabetes	75 433 (21.1)	0*
Chronic renal failure	20 349 (5.7)	0*
Hypercholesterolaemia	112 713 (31.5)	0*
Hypertension	174 596 (48.9)	0*
Previous or current smoker	254 215 (71.2)	0*
Asthma or COPD	52 030 (14.6)	0*
Family history of CHD	72 444 (20.3)	0*
Presenting characteristics		
Systolic blood pressure, mm Hg, mean (SD)	142.5 (28.4)	59 962 (16.8)
Systolic blood pressure, <90 mm Hg	7280 (2.5)	59 962 (16.8)
Heart rate, bpm, mean (SD)	80 (67–95)	59 177 (16.6)
Heart rate >110 bpm	32 964 (11.1)	59 177 (16.9)
Creatinine; mean (SD)	92 (76–114)	147 959 (41.4)
Troponin elevation	321 212 (94.6)	17 559 (4.9)
Cardiac arrest	6178 (1.8)	21 038 (5.9)
ECG		
No acute changes	51 214 (15.7)	31 825 (8.9)
ST-segment elevation	14 336 (4.4)	
Left bundle branch block	21 149 (6.5)	
ST-segment depression	84 821 (26.1)	
T-wave changes only	85 474 (26.3)	
Other acute abnormality	68 409 (21.0)	
Use of a loop diuretic	89 438 (30.2)	61 294 (17.1)
GRACE risk score category		
Low (≤88)	25 787 (18.2)	215 599 (60.4)
Intermediate (88–110)	38 897 (27.5)	
High (>110)	76 945 (54.3)	

GRACE risk score category as defined by NICE.

*Missing data default imputed to ‘No’.

CABG, coronary artery bypass graft; CHD, coronary heart disease; COPD, chronic obstructive pulmonary disease; IMD, Index of Multiple Deprivation; NICE, National Institute for Health and Care Excellence; NSTEMI, non-ST-segment elevation myocardial infarction; PCI, percutaneous coronary intervention.

Over half (n=254 215, 71.2%) were previous or current smokers, 48.9% (n=174 596) had hypertension, 21.1% (n=75 433) diabetes and 14.6% (n=52 030) had asthma or COPD. Over 2% (n=7280) of patients had an admission systolic blood pressure <90 mm Hg. About half (n=184 631, 56.8%) of all electrocardiographic changes were ST-segment deviation or T-wave inversion with 15.7% (n=51 214) of patients having no acute changes. According to the mini-GRACE risk score, 8 in 10 patients were in intermediate or high risk. The distribution of patients eligible to receive guideline-indicated treatments is shown in table 2, the highest being for an ECG and the lowest for smoking cessation advice.

**Table 2:** Eligibility and receipt of guideline-indicated interventions, NSTEMI, 2003–2013

Guideline-indicated intervention	Number (%) of NSTEMI who received a guideline-indicated intervention	Number of NSTEMI eligible for a guideline-indicated intervention
ECG	336 094 (94.1)	357 228
Acute aspirin	212 837 (88.7)	239 876
Echocardiography	178 851 (50.1)	357 195
Coronary angiography	196 781 (57.4)	342 856
Aspirin at discharge	279 584 (89.1)	313 901
P2Y_12_ inhibitors	121 427 (41.0)	296 450
ACEis/ARBs	81 176 (67.9)	119 625
β Blockers	80 600 (74.8)	107 698
Statins at discharge	275 626 (86.2)	319 747
Aldosterone antagonists	134 (23.7)	566
Dietary advice	111 759 (32.6)	342 960
Smoking cessation advice	31 683 (12.5)	254 215
Cardiac rehabilitation	257 875 (76.7)	336 146

ACEis, ACE inhibitors; ARB, angiotensin receptor blockers.

### Guideline-indicated interventions

The proportion of NSTEMI who received optimal care was low (48 257/357 228, 13.5%). One in 10 (n=42 229, 11.8%) received ≤40% of the guideline-indicated treatments for which they were eligible, 6 in 10 (n=208 930, 58.5%) received >40% to ≤79% and 3 in 10 (n=106 069, 29.7%) received >79%. The most frequently missed were dietary advice (n=231 201, 67.4%), smoking cessation advice (n=222 532, 87.5%), echocardiography (n=178 344, 49.9%), P2Y_12_ inhibitors at discharge from hospital (n=175 023, 59.0%), coronary angiography (n=146 075, 42.6%) and in-hospital aspirin (n=97 411, 44.8%) (table 2). Over half of the patients (n=207 355, 58.1%) were not under the care of a cardiologist.

### Geographic variation

**Figure 2:**
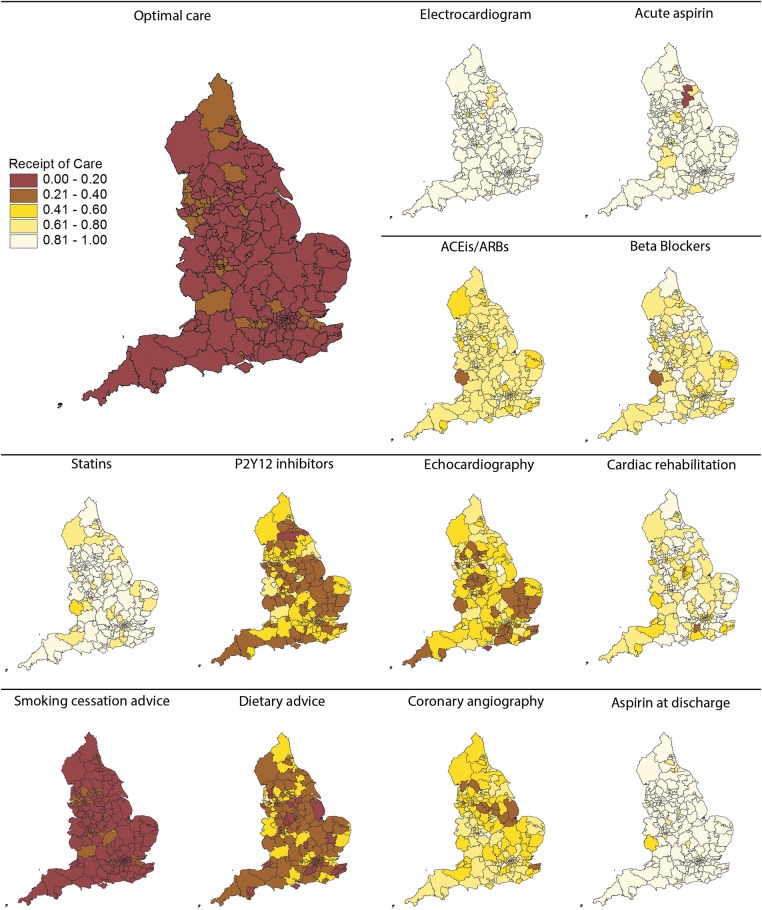
Geographic variation proportions of eligible patients who received guideline-indicated interventions, for each intervention and for optimal care, by CCG. ACEis, ACE inhibitors; ARB, angiotensin receptor blockers; CCGs, Clinical Commissioning Groups.

For CCGs, the proportion of patients who received optimal care was low (median 12.8%, IQR 0.7–18.1%) (figure 2). The greatest variation in care was for aldosterone antagonists (median 16.7%, IQR 0.0–40.0%) and least for use of an ECG (96.7%, 92.5–98.7%). High rates of the prescription of aspirin acutely (median 92.8%, IQR 88.6–97.1%), aspirin at discharge from hospital (90.1%, 85.1–93.3%) and statins (86.4%, 82.3–91.2%) were consistent. The provision of echocardiography (50.3%, 38.3–61.9%), cardiac rehabilitation (79.7%, 68.2–87.1%), coronary angiography (57.4%, 48.8–66.7%), the prescription of ACEis/ARBs (69.0%, 63.6–74.0%) and β blockers (76.3%, 70.4–82.0%) was intermediate and varied widely, while the provision of smoking cessation advice (11.6%, 8.7–16.6%), dietary advice (32.4%, 23.9–41.7%) and P2Y_12_ inhibitors (39.7%, 32.4–46.9%) was poor.

Across SCNs, the proportion of patients who received optimal care was also low (median 12.2%, IQR 11.5–15.9%) (see online supplementary table S4). The area with the highest proportion of patients who received optimal care was North East and North Cumbria (n=7045, 20.0%), and the lowest was the East Midlands (n=3409, 10.3%). Rates of guideline-indicated interventions were intermediate-to-high and varied little between SCNs for ECG (median 95.0%, IQR 92.0–96.0%), acute aspirin (91.0%, 88.0–92.0%), statins (86.0%, 84.0–87.0%), aspirin on discharge (89.0%, 87.0–90.0%), cardiac rehabilitation (79.0, 72.0–82.0%), β blockers (76.0%, 73.0–76.0%) and the prescription of ACEis/ARBs (68.0%, 67.0–70.0%). Performance was consistently low across SCNs for P2Y_12_ inhibitors (40.0%, 39.0–42.0%), aldosterone antagonists (27.0%, 20.0–28.0%), smoking cessation advice (13.0%, 12.0–17.0%) and dietary advice (32.0%, 28.0–37.0%). Echocardiography (50.0%, 45.0–55.0%) and coronary angiography (58.0%, 52.0–61.0%) were provided at an intermediate rate.

### Variance components

**Figure 3:**
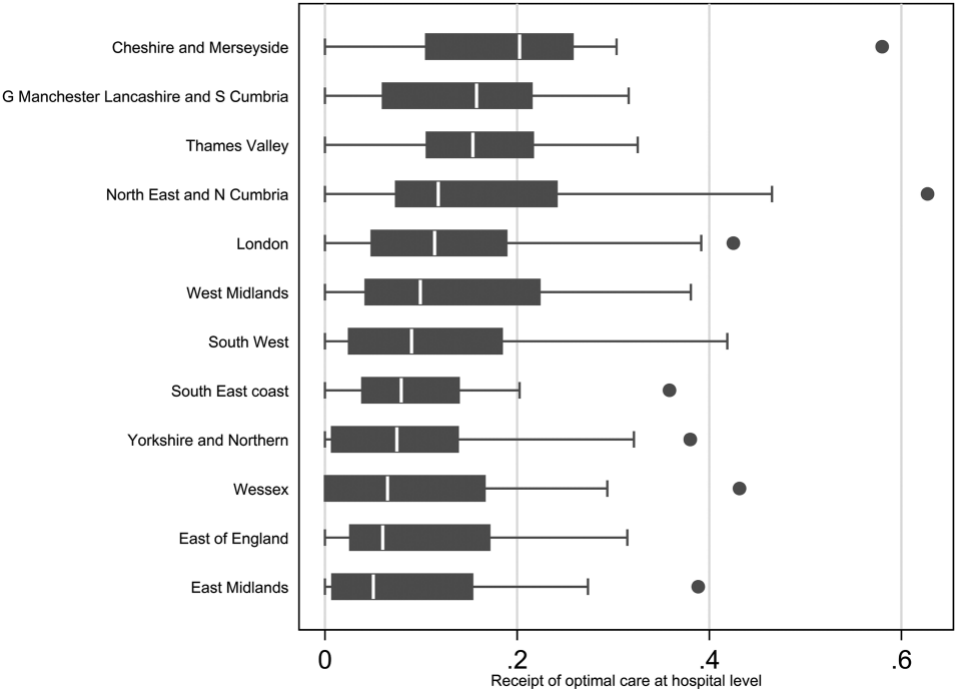
Optimal care variation in hospitals by SCNs. SCNs, Strategic Clinical Networks.

The between-unit variance, standardised for case mix, was low for SCNs (0.004, 95% CI 0.0004 to 0.03) and CCGs (0.004, 0.001 to 0.03) but much higher for hospitals (1.92, 95% CI 1.51 to 2.44). Moreover, the model indicated that 0.2% of the remaining variation in the provision of guideline-indicated care after case mix adjustment was between SCNs (ICC 0.002, 95% CI 0.0002 to 0.01) and 0.2% between CCGs (ICC 0.002, 95% CI 0.0007 to 0.01) with 99.6% between hospitals (ICC 0.996, 95% CI 0.976 to 0.999) (table 3). Hospital variation in optimal care was consistently wide within SCNs of differencing performance (figure 3).

**Table 3:** Parameter estimates, p values, SEs and 95% CIs for optimal receipt of care for the Poisson model

Fixed effects	Incidence ratios	p Value	95% CI
Sex (male vs female)	1.12	<0.001	1.11 to 1.15
Deprivation according to IMD score			
1 (least deprived)	1	–	1
2	0.98	0.34	0.95 to 1.02
3	0.99	0.41	0.95 to 1.02
4	0.97	0.06	0.93 to 1.00
5 (most deprived)	0.96	0.02	0.92 to 0.99
Ethnicity			
White	1	–	1
Black	0.99	0.78	0.90 to 1.08
Asian	1.02	0.32	0.98 to 1.07
Mixed	1.21	0.07	0.98 to 1.48
Other	0.92	0.10	0.84 to 1.02
GRACE risk score category			
Low (≤88)	1	–	1
Intermediate (88–110)	0.97	0.16	0.94 to 1.01
High (>110)	0.78	<0.001	0.76 to 0.81
Current smoker (Yes vs No)	1.16	<0.001	1.14 to 1.19
Prior diabetes (Yes vs No)	0.99	0.88	0.98 to 1.02
Prior MI (Yes vs No)	0.90	<0.001	0.88 to 0.92
Prior angina (Yes vs No)	0.91	<0.001	0.89 to 0.93
Prior PCI (Yes vs No)	0.98	0.33	0.95 to 1.02
Prior CABG (Yes vs No)	0.95	0.01	0.92 to 0.99
Prior peripheral vascular disease (Yes vs No)	0.95	0.03	0.91 to 0.99
Hypercholesterolemia	1.11	<0.001	1.08 to 1.13
Prior hypertension (Yes vs No)	1.02	0.08	1.00 to 1.04
Prior cerebrovascular disease (Yes vs No)	0.88	<0.001	0.84 to 0.90
Prior chronic obstructive pulmonary disease/asthma (Yes vs No)	0.94	<0.001	0.92 to 0.97
Family history of CHD (Yes vs No)	1.15	<0.001	1.13 to 1.17
Year	1.60	<0.001	1.60 to 1.62
**Random effects**	**Variance**	**Standard error**	**95% CI**
Hospital variance			
	1.92	0.24	1.51 to 2.44
CCG variance			
	0.004	0.004	0.001 to 0.03
SCN variance			
	0.004	0.004	0.0004 to 0.03

CABG, coronary artery bypass graft; Clinical Commissioning Groups (CCGs); CHD, coronary heart disease; COPD, chronic obstructive pulmonary disease; IMD, Index of multiple deprivation; PCI, percutaneous coronary intervention; Strategic Clinical Networks (SCNs).

### Temporal changes

Table 4 shows the improvement in the provision NSTEMI care from 2003/2004 to 2012/2013, being most pronounced for coronary angiography (median CCG rates: 33 vs 83%), ACEis/ARBs (71 vs 100%) and β blockers (77 vs 100%). Even so, there was only a modest improvement in optimal care, and although the correlation between care in CCGs over the study period was significant, it was weak (ρ=0.36, p<0.001). Temporal trends in the proportion of NSTEMI who received guideline-indicated treatments between 2003 and 2013 are shown in interactive figures 4 and 5 which can be accessed by clicking on the following web links: http://www.personal.leeds.ac.uk/~medcardp/googleplots.html and http://www.personal.leeds.ac.uk/~medcardp/map.html respectively.

**Table 4:** Temporal changes in the proportion of NSTEMI receiving guideline-indicated treatments, 2003/2004 vs 2012/2013 in CCGs

Guideline-indicated intervention	Biennial year	
	2003/2004 Median (IQR)	2012/2013 Median (IQR)
Optimal care	0.00	0.34 (0.23–0.46)
ECG	0.86 (0.69–0.96)	1.00 (0.99–1.00)
Acute aspirin	0.88 (0.81–0.94)	0.97 (0.93–0.99)
ACEis/ARBs	0.71 (0.65–0.76)	1.00 (1.00–1.00)
β Blockers	0.77 (0.71–0.83)	1.00 (0.93–1.00)
Statins	0.83 (0.77–0.88)	0.95 (0.91–0.98)
P2Y_12_ inhibitors	0.00	0.94 (0.88–0.98)
Aldosterone antagonist	–	0.00 (0.00–1.00)
Echocardiography	0.41 (0.27–0.57)	0.63 (0.51–0.76)
Cardiac rehabilitation	0.73 (0.60–0.83)	0.87 (0.74–0.94)
Smoking cessation advice	0.00	0.69 (0.47–0.87)
Dietary advice	0.00	0.84 (0.62–0.93)
Coronary angiography	0.33 (0.21–0.47)	0.83 (0.75–0.89)
Aspirin on discharge	0.89 (0.83–0.93)	0.97 (0.94–0.99)

Median represents the median of the proportion of eligible NSTEMI who received guideline-indicated care. ACEis, ACE inhibitors; ARB, angiotensin receptor blockers; CCGs, Clinical Commissioning Groups; NSTEMI, non-ST-segment elevation myocardial infarction.

### Excess deaths

Over the study period, the case mix standardised excess mortality associated with non-receipt of optimal guideline-indicated care varied between hospitals (median number of deaths 39, IQR 15–62) between 2003 and 2013.

## Discussion

Over a 10-year study period, we found evidence for widespread suboptimal use of guideline-indicated treatments for the management of NSTEMI. While the use of specific treatments for NSTEMI, such as pharmacological therapies and invasive coronary procedures, varied between CCGs, most of the variation (after accounting for differences in patients) was explained by differences in the provision of care by hospitals. We found that the geographical variation in NSTEMI treatments was associated with geographical variation in the number of excess deaths. Together, the findings from our study suggest that there is substantial scope to improve the provision and uniformity of NSTEMI care across the NHS and, therefore, reduce premature cardiovascular death.

In contrast to recent reports of geographic and temporal variation in the inappropriate use of cardiac procedures,^33^ we found that many patients who were eligible to receive care did not. The greatest variation was for the prescription of aldosterone antagonists and least for use of an ECG. Specifically, when we defined care using a composite score according to eligibility for any of the 13 international guideline recommended treatments and according to the date from which they were published, we found that optimal care was delivered infrequently. Even though we found that optimal care varied geographically, it was only when we evaluated specific interventions that we found much wider variations in care. This was evidenced by wide variation in the provision of key interventions such as coronary angiography, cardiac rehabilitation and pharmacological therapies.

After adjustment for case mix, most of the variation in NSTEMI care occurred at the level of the hospital and to a much lesser extent between CCGs and SCNs. This finding is not surprising because hospitals are the service providers for the treatment of NSTEMI. Our earlier research has described the missed opportunities for care among patients who present to NHS hospitals with acute myocardial infarction and that this was significantly associated with reduced survival.^11,13^ We have also shown that between-hospital variation in care is wider in the UK than in Sweden, and this was also associated with a higher and wider range of mortality rates in the UK.^2^ For this study, we elected to investigate geographic variation in care according to CCGs rather than hospitals because CCGs are central to the contracting of NSTEMI services and to whom hospitals are financially accountable.

Causes of healthcare variation are numerous and complex. They may be due to differences in patterns of illness, clinicians' behaviour or the effects of incentives in the financing of healthcare.^34^ ^35^ In this study, we found that variation in the provision of NSTEMI treatment remained after adjusting for patient sociodemographic and clinical characteristics. This suggests that modifiable factors such as procurement, infrastructure, availability of specialist services and physician education are critical.^34^ Typically the use and availability of cardiac procedures are closely related.^15^ ^36^ However, this is not always the case and it is possible that other factors are also at play such as physician-dependent risk-aversion to invasive cardiac care,^37^ a perception that higher risk patients do not have a net benefit from NSTEMI care, difficulties in obtaining an early and accurate diagnosis of NSTEMI,^38^ ^39^ the availability of specialist cardiac, emergency and ambulance services staff, size and type of acute hospital^40^ as well as the placement patients with NSTEMI on adequately staffed specialist wards within a hospital.^37^ In addition, we found little evidence to suggest that the performance of a geographic unit remained constant (though overall there was improvement in care over time). Our findings suggest that regional networks of care for NSTEMI are immature and can be compared with the provision of STEMI care in the UK where there is institutional (and regional) operationalisation effecting high-quality care and low mortality rates.^40^ ^41^

Ours and others' previous work have demonstrated significant associations between adherence to evidence-based care for the management of NSTEMI and better clinical outcomes.^11^ ^26^ ^42^ Data from the CRUSADE registry have also shown that patients with NSTEMI who receive guideline-indicated care have better outcomes and that this is associated with the type of hospital to which a patient is admitted.^13^ ^43^ Even though our research concentrated mainly the evaluation of processes of care, we also found that there was variation between hospitals in the numbers of potentially avoidable deaths. This is not a surprising finding because our study was of guideline-indicated treatments endorsed by international societies with mostly Class 1A recommendations that have been shown in randomised studies to improve clinical outcomes.^8^ ^44^ Tackling inequalities in care at the level of the healthcare professional, service provider and commissioner will lead to a reduction in the numbers of deaths from NSTEMI.

By representing processes of clinical care at the level of the CCG, commissioners may identify where and what service may require closer attention. Moreover, it is plausible that the introduction of a performance-based tariff for NSTEMI (or an additional best practice payment)^45^ may improve outcomes and reduce provider variation. This is because others have reported associations between performance-based commissioning and improved quality of care and outcomes, albeit not for NSTEMI.^46–49^ For example, the introduction of the Advancing Quality programme across all NHS hospitals in the north-west of England was associated with a significant reduction in combined short-term mortality for pneumonia, heart failure and acute myocardial infarction.^49^

Our study has strengths in that it evaluated care across a national healthcare service and accesses a clinical registry designed specifically to evaluate quality of NSTEMI care. Even though variance in adherence to guideline-indicated care for NSTEMI has been reported by others,^50^ ^51^ it has not been evaluated across a single healthcare system—which should, therefore, vary to a lesser degree than across different health systems operating in one country. In this study, we undertook a systematic approach to evaluate variation in care.^52^ First, we quantified variability in rates across different layers of geographic units. Second, we calculated indexes, including the systematic component of variation. Third, we developed explanations for the variation by adjusting for case mix. In addition to the main findings, our study is an example of how patient-level clinical registries allow higher resolution interrogation of pathways of care,^53^ the results of which should stimulate bespoke quality improvement tailored to region and intervention.

Our study, however, has limitations. MINAP does not collect all cases of NSTEMI—even so, our study was designed to study the impact of missed care at the level of the patient and not the numbers of NSTEMI hospitalised. We speculate that MINAP captures less than half of all NSTEMI; consequently, the number of missed opportunities that we report will be underestimated. Conversely, it is possible that some patients will have received treatments, but this not recorded in MINAP. We used CCGs to investigate consistency in geographic unit performance over time, when they only recently have replaced Primary Care Trusts and may not have the same Cartesian boundaries. The deficits in care for smoking cessation and dietary advice may be artificially inflated because advice about smoking and diet are implicit in cardiac rehabilitation programmes and there may have been preferencing by coders towards recording cardiac rehabilitation.

In conclusion, this study found that between 2003 and 2013, most of the 357 228 patients hospitalised with NSTEMI did not receive optimal international guideline-indicated care, although this finding was less evident in the latter years of study. Receipt of optimal care for the management of NSTEM, and more so the individual components of the NSTEMI treatment pathway, varied widely by hospitals across the English NHS and was associated with between-hospital variation in excess deaths. Given adherence to guideline-indicated care for the management of NSTEMI is associated with improved clinical outcomes, addressing the provision of care through performance-based commissioning and stronger networks of NSTEMI care has the potential to reduce premature deaths from cardiovascular disease.
