# Supplementary material for: Geographic variation in the treatment of non-ST-segment myocardial infarction in the English National Health Service: a cohort study
Source: BMJ Open. 2016 Jul 12;6(7):e011600. doi: 10.1136/bmjopen-2016-011600 (PMC4947744; doi:10.1136/bmjopen-2016-011600)
Supplement: Supplementary data [file bmjopen-2016-011600supp.pdf]

Supplementary file

Section 1: Evidence based care interventions

Mapping of the European Society of Cardiology guidelines for the management of NSTEMI and the ESC Expert Consensus Documents to MINAP data fields

Figure S1 and Table S1 show the corresponding European Society of Cardiology (ESC) guidelines for the management of NSTEMI mapped to MINAP. The guidelines in red, for example anticoagulation medication and glycoprotein IIb/IIIa (figure S1), were excluded from the analysis because of inadequate data quality or recording. Year of recommendation of each care intervention (according to the ESC publications) for each care opportunity was taken into consideration when determining each patient's eligibility to receive care.[1-5] Patients were also classed as ineligible for one or more care opportunity if the treatment was contra-indicated, not indicated, not applicable, or if the patient declined treatment as recorded in MINAP.

Figure S1: European Society of Cardiology guidelines for the management of NSTEMI, 1 care interventions and corresponding MINAP data availability

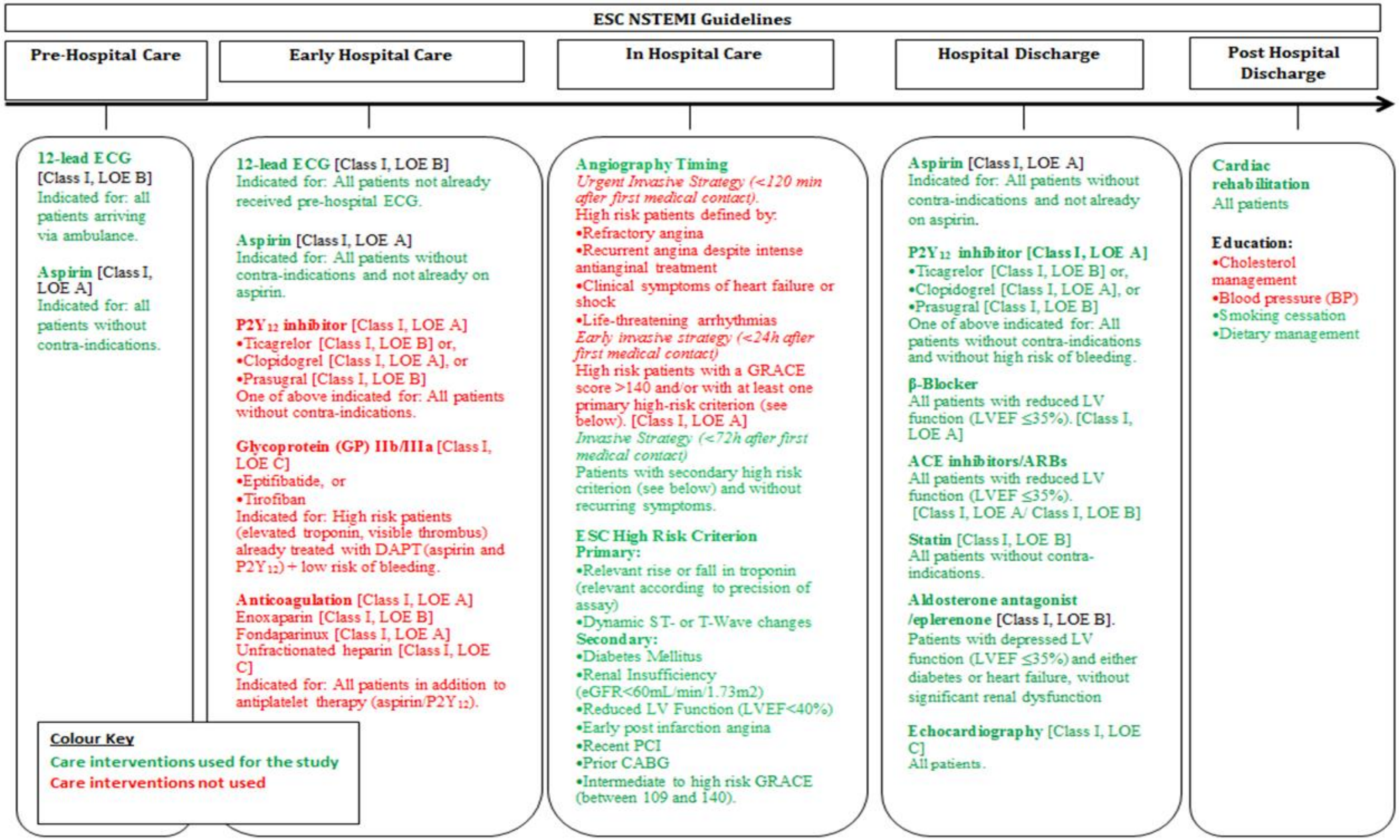

Table S1: Mapping of corresponding ESC guidelines for the management of NSTEMI to MINAP data fields.

**Care Pathway Components**	**Eligibility Criteria**	**MINAP variable**
12 lead electrocardiogram (ECG)		
Pre-Hospital	All patients arriving via ambulance	Place were electrocardiogram (ECG) was taken
In-Hospital	All patients not already received ECG pre-hospital	ECG appearance or ECG place
Aspirin		
Pre-Hospital	All patients arriving via ambulance and not already on aspirin or contraindicated	Where aspirin was given
In-Hospital	All patients not already on aspirin or contraindicated	Where aspirin was given
At Discharge	All patients not already on aspirin or contraindicated	Discharged on aspirin
$P2Y_{12}$ inhibitor		
At Discharge	All patients not contraindicated and without high risk of bleeding	Discharged on thienopyridine or discharged on ticagrelor
Coronary angiography	All non-high risk patients unless contraindicated<br><br>Patients with one or more secondary high risk criterion as defined in Figure S1	Coronary Angiography irrespective of the time they received from admission<br><br>If time from first medical contact to angiography is <72 hours

β Blocker	All patients with reduced LV function and no contraindications.	Discharged on beta blocker<br><br>If given oral beta blocker and if discharged on beta blocker
ACE inhibitors/ARBs	All patients with reduced LV function and no contraindications.	Discharged on ACEi or discharged ARB
Statin	All patients unless contraindicated	Discharged on statin
Aldosterone antagonist/eplerenone	Patients with depressed LV function (LVEF ≤35%) and either diabetes or heart failure, without significant renal dysfunction and already treated with ACE inhibitors and beta blockers	Discharged aldosterone
Echocardiography	All patients unless not indicated.	Stress echocardiography or echocardiography
GRACE Risk Score Recorded in Data (i.e. before our own derivation/calculation).	All patients	
Referral for Cardiac Rehabilitation	All patients eligible unless not indicated	Cardiac rehabilitation
Smoking Cessation Advice	All patients eligible unless not applicable	Smoking cessation
Dietary Advice	All patients eligible if applicable.	Dietary advice

Section 2: Multiple Imputation

Multiple imputations by chained equations (MICE) [6] were used to create 10 imputed datasets for missing data for all components of the GRACE risk score and other patient demographic variables. A default imputation (missing data default imputed to "NO") strategy based on clinical expert opinion was implemented for cardiovascular history, cardiovascular risk factors, and categorical treatment variables. [6] The imputation models were based on previous work and we ensured consistency with the accelerated failure time (AFT) survival model by including the Nelson-Aalen estimate for survival and a censoring indictor in the imputation model. The full imputation model used is defined in detail in table S2. Predictive mean matching was used for continuous variables with nonlinear associations.

Table S2: Imputation Strategy

**Variable**	**Variable Type**	**Missing (%)**	**Imputation method**
ECG appearances on which treatment was based	Categorical	9.2	Polytomous regression
Cardiac arrest	Binary	5.9	Logistic regression
Uncensored peak troponin measurement in ng/ml	Continuous	4.9	Predictive mean matching
Age	Continuous	0.2	Predictive mean matching
Systolic blood pressure	Continuous	17.1	Predictive mean matching
Heart rate	Continuous	16.9	Predictive mean matching
Loop diuretic used	Binary	17.4	Logistic regression
Creatinine level	Continuous	42.6	Predictive mean matching
Ethnicity	Categorical	9.8	Polytomous regression
Sex	Binary	0.2	Logistic regression
Index of multiple deprivation score	Continuous	7.8	Predictive mean matching
Derived identification	Continuous	0	Predictor/ Auxiliary /Partially Observed
Latent classes	Categorical	0	Predictor/ Auxiliary /Partially Observed
Cumulative receipt of care	Continuous	0	Predictor/ Auxiliary /Partially Observed
Cumulative receipt of care	Binary	0	Predictor/ Auxiliary /Partially Observed
Arrival year	Continuous	0	Predictor/ Auxiliary /Partially Observed

Nelson-Aalen survival estimate	Continuous	0	Predictor/ Auxiliary /Partially Observed
Censoring indicator	Binary	0	Predictor/ Auxiliary /Partially Observed
Previous myocardial infarction	Binary	8.0	Predictor/ Auxiliary and Default imputed
Previous angina	Binary	8.9	Predictor/ Auxiliary and Default imputed
Hypercholesterolaemia	Binary	11.0	Predictor/ Auxiliary and Default imputed
Previous hypertension	Binary	8.3	Predictor/ Auxiliary and Default imputed
Peripheral vascular disease	Binary	11.5	Predictor/ Auxiliary and Default imputed
Cerebrovascular disease	Binary	10.6	Predictor/ Auxiliary and Default imputed
Chronic obstructive pulmonary disease/Asthma	Binary	11.4	Predictor/ Auxiliary and Default imputed
Congestive cardiac failure	Binary	10.4	Predictor/ Auxiliary and Default imputed
Previous percutaneous coronary intervention	Binary	10.1	Predictor/ Auxiliary and Default imputed
Previous coronary artery bypass graft	Binary	9.8	Predictor/ Auxiliary and Default imputed
Smoker ever	Binary	7.5	Predictor/ Auxiliary and Default imputed
Diabetes	Binary	7.1	Predictor/ Auxiliary and Default imputed
Family history of chronic heart disease	Binary	37.3	Predictor/ Auxiliary and Default imputed
Care by Cardiologist	Binary	39.1	Predictor/ Auxiliary and Default imputed

Table S3: Adjusted mortality risk

Treatment	Complete case analysis<br>Adjusted TRs (95% CI)	Multiple imputation analyses<br>Adjusted TRs (95% CI)	P-value
Optimal care	1	1	-
Sub-optimal care§	0.40 (0.38, 0.43)	0.44 (0.41, 0.45)	< 0.001

Section 3: Avoidable deaths

The number of preventable deaths comparing patients who received optimal treatment with those who received sub-optimal treatment was calculated using a method described in Ford et al (2007). [7] The risk associated with sub-optimal treatment (adjusted time ratios of these effects (table S3), (a)), was multiplied by the proportion of patients who had received sub-optimal treatment (proportion of the population at risk (b)) per hospital. The product was then multiplied by the 12-month mortality rate of the patients in the sub-optimal treatment group (c) per hospital. The figure obtained was then multiplied by the total number of NSTEMI presentations (d) from 2003 – 2013 per hospital. The formula to determine the preventable deaths is shown below:

$$a \times b \times c \ \times d$$

For example from the study; we found that sub-optimal treatment shortened time to death by 56% (a=0.44). For one of the hospitals 22.6% of the cohort received sub-optimal treatment (b=0.23). The 12-month mortality rate of the patients in the sub-optimal treatment group for the same hospital was 21.8% (c=0.22) and the total number of NSTEMI admissions from 2003-2013 for the hospital was 1,769 (d=1,769); the estimated number of potentially preventable deaths for the hospital would be:

$\left((0.44 \times 0.23)\right) \times 0.22 \ \times 1{,}769$ =39 deaths.

Table S4: Proportions of patients (of the eligible) receiving each care intervention by SCNs

	**GEOGRAPHIC REGION (SCNs)**											
**Treatments** n (%)	**Cheshire and Merseyside**	**East Midlands**	**East of England**	**G Manchester Lancashire and S Cumbria**	**London**	**North East and N Cumbria**	**South East coast**	**South West**	**Thames Valley**	**Wessex**	**West Midlands**	**Yorkshire and Northern**
**Electrocardiogram**	18,412 (94.3)	32,080 (96.7)	45,553 (95.9)	26,346 (88.8)	32,524 (94.6)	34.149 (96.9)	24,522 (95.8)	27,612 (94.6)	9,236 (94.9)	14,903 (93.1)	28,563 (90.7)	42,194 (92.3)
**Acute aspirin**	11,921 (91.6)	18,412 (92.3)	30,302 (91.5)	19,993 (90.5)	20,143 (90.3)	18,733 (88.4)	16,291 (92.6)	17,045 (90.0)	6,034 (94.2)	10,385 (95.1)	20,103 (85.3)	23,475 (76.3)
**ARB/ACEi**	3,577 (64.5)	7,522 (68.1)	10,670 (66.8)	6,746 (70.6)	7,654 (70.1)	8,440 (64.4)	5,667 (67.3)	6,824 (66.3)	2,402 (72.3)	4,333 (73.7)	6,892 (70.0)	10,449 (67.7)
**Beta blockers**	3,252 (68.9)	7,849 (74.7)	10,046 (72.7)	6,834 (76.2)	7,543 (75.5)	8,720 (73.1)	5,798 (75.9)	6,612 (71.5)	2,367 (76.3)	4,089 (78.9)	7,032 (76.4)	10,458 (78.3)
**Statins**	13,292 (84.9)	25,153 (84.9)	35,808 (84.4)	24,896 (90.6)	27,022 (86.7)	25,154 (84.4)	20,574 (86.2)	22,005 (83.2)	8,060 (88.5)	12,260 (88.1)	24,513 (87.4)	36,889 (87.4)
**$P2Y_{12}$ inhibitors**	5,564 (39.3)	11,180 (39.9)	15,646 (39.1)	11,836 (42.3)	12,388 (42.3)	11,570 (43.2)	9,388 (41.5)	9,863 (41.4)	3,944 (46.7)	4,599 (40.0)	10,468 (40.4)	14,981 (38.4)
**Aldosterone antagonists**	8 (33.3)	9 (18.4)	15 (27.3)	10 (33.3)	20 (27.4)	19 (18.1)	12 (28.6)	7 (20.0)	5 (31.3)	6 (14.6)	12 (21.1)	11 (28.2)
**Echocardiography**	11,918 (61.0)	17,252 (52.0)	21,268 (44.8)	15,938 (53.7)	19,453 (56.6)	20,788 (59.0)	10,666 (41.7)	14,001 (50.0)	4,524 (46.5)	8,850 (55.3)	14,570 (46.3)	19,623 (42.9)
**Cardiac rehabilitation**	14,120 (74.2)	22,050 (71.8)	35,708 (81.4)	21,464 (77.7)	21,295 (65.1)	26,916 (78.8)	17,418 (71.8)	17,147 (64.9)	7,029 (79.1)	13,443 (87.0)	24,180 (81.9)	37,105 (85.4)
**Smoking cessation advice**	2,215 (17.0)	2,888 (11.8)	3,310 (9.3)	3,396 (17.2)	2,983 (11.5)	3,709 (14.6)	2,052 (11.7)	2,033 (9.9)	848 (13.1)	1,227 (10.5)	2,515 (11.5)	4,507 (14.1)

**Dietary advice**	7,488 (39.6)	8,214 (26.3)	16,187 (34.9)	10,946 (38.7)	8,592 (26.1)	12,627 (36.9)	7,632 (31.3)	7,721 (27.9)	3,510 (38.7)	4,980 (31.8)	9,712 (33.0)	14,150 (31.6)
**Coronary angiography**	8,569 (47.6)	15,379 (47.8)	26,371 (57.7)	14,618 (51.3)	23,269 (69.6)	17,773 (52.6)	14,718 (59.6)	16,869 (61.3)	5,741 (59.9)	10,493 (66.1)	20,370 (67.0)	22,611 (52.3)
**Aspirin on discharge**	12,887 (86.9)	25,395 (87.3)	36,255 (87.4)	24,797 (93.2)	27,371 (88.8)	26,056 (88.0)	21,060 (90.1)	23,441 (89.8)	8,179 (89.9)	12,940 (91.5)	24,450 (88.6)	36,753 (89.3)
**Optimal care**	3,099 (15.9)	3,409 (10.3)	5,498 (11.6)	5,128 (17.3)	4,194 (12.2)	7,045 (20.0)	3,133 (12.2)	3,342 (11.5)	1,757 (18.0)	2,386 (14.9)	4,356 (13.8)	4,910 (10.7)

References

1. Hamm C, Bassand J, Agewall S, et al. ESC Committee for Practice Guidelines. ESC Guidelines for the management of acute coronary syndromes in patients presenting without persistent ST-segment elevation: The Task Force for the management of acute coronary syndromes (ACS) in patients presenting without persistent ST-segment elevation of the European Society of Cardiology (ESC). Eur Heart J 2011;**32**(23):2999-3054
2. López-Sendó J, Swedberg K, McMurray J, et al. Expert consensus document on β-adrenergic receptor blockers. Eur Heart J 2004;**25**(15):1341-62
3. Erhardt L, Herlitz J, Bossaert L, et al. Task force on the management of chest pain. Eur Heart J 2002;**23**(15):1153-76
4. Bassand J, Hamm CW, Ardissino D, et al. Guidelines for the diagnosis and treatment of non-ST-segment elevation acute coronary syndromes. Eur Heart J 2007;**28**(13):1598-660
5. Bertrand M, Simoons M, Fox K, et al. Management of acute coronary syndromes: acute coronary syndromes without persistent ST segment elevation. Recommendations of the Task Force of the European Society of Cardiology. Eur Heart J 2000;**21**(17):1406-32
6. Cattle B, Baxter P, Greenwood D, Gale C, West R. Multiple imputation for completion of a national clinical audit dataset. Stat Med 2011;**30**(22):2736-53
7. Ford E, Ajani U, Croft J, et al. Explaining the decrease in US deaths from coronary disease, 1980–2000. N Engl J Med 2007;**356**(23):2388-98
